# The Changes in mGluR2 and mGluR7 Expression in Rat Medial Vestibular Nucleus and Flocculus Following Unilateral Labyrinthectomy

**DOI:** 10.3390/ijms141122857

**Published:** 2013-11-20

**Authors:** Wen Zhou, Liu-Qing Zhou, Su-Lin Zhang, Bo Liu, Yang-Ming Leng, Ren-Hong Zhou, Wei-Jia Kong

**Affiliations:** 1 Department of Otorhinolaryngology, Union Hospital, Tongji Medical College, Huazhong University of Science and Technology, 1277 Jiefang Avenue, Wuhan 430022, China; E-Mails: zhouwen_124@163.com (W.Z.); zlq1985319@gmail.com (L.-Q.Z.); zhang_lilac@sina.com (S.-L.Z.); borliu@gmail.com (B.L.); lyangming@gmail.com (Y.-M.L.); yoyochow@126.com (R.-H.Z.); 2 Institute of Otorhinolaryngology, Union Hospital, Tongji Medical College, Huazhong University of Science and Technology, 1277 Jiefang Avenue, Wuhan 430022, China; 3 Key Laboratory of Neurological Disorders of Education Ministry, Tongji Medical College, Huazhong University of Science and Technology, Wuhan 430022, China

**Keywords:** flocculus, medial vestibular nucleus, mGluR2, mGluR7, vestibular compensation

## Abstract

It is known that the medial vestibular nucleus (MVN) and the cerebellar flocculus are the key areas, which contribute to the behavioral recovery (“vestibular compensation”) after unilateral labyrinthectomy (UL). In these areas, how the genetic activities of the metabotropic glutamate receptors mGluR2 and mGluR7 performance after UL is unknown. With the means of quantitative real-time PCR, Western blotting, and immunohistochemistry, we analyzed the expression of mGluR2 and mGluR7 in the bilateral MVN and the flocculus of rats in different stages after UL (the 1st, 3rd, and 7th day). Our results show that in the MVN, the mRNA, and protein expressions of mGluR7 were ipsilaterally decreased at the 1st day following UL. However, in the MVN, no change was observed in the mRNA and protein expressions of mGluR2. On the other hand, the mRNA and protein expression of mGluR2 were enhanced in the ipsilateral flocculus at the 1st day following UL, while in the flocculus no change was shown in mGluR7 mRNA and protein expressions. Our results suggest that mGluR2 and mGluR7 may contribute to the early rebalancing of spontaneous resting activity in the MVN.

## 1. Introduction

Vestibular compensation is a process of the plasticity of central nervous system (CNS). Traditionally, unilateral labyrinthectomy (UL) has been used as a model for investigating the post-lesional plasticity of vestibular nuclei complex in adult animals [1]. With UL, the deafferented second-order neurons lose their normally high resting activity, while the contralateral neurons become hyperactive. The consequent asymmetry of the resting discharge between the intact and deafferented vestibular nucleus complex (VNC) is thought to induce the severe oculo-motor and postural symptoms shortly following UL [2–5]. These symptoms rapidly disappear when vestibular compensation (VC) occurs [6]. Most current theories of VC assume that the initial drastic asymmetry in the resting discharge of medial vestibular nucleus (MVN) neurons is due to a large imbalance in the reciprocal commissural inhibitory system that links the MVN of the two sides and recovery of resting activity in the deafferented ipsi-lesional MVN neurons partly by counteracting this increased commissural inhibition after UL [7–9]. As lesions of the ipsilateral vestibular nucleus (ipsi-VN) can prevent static compensation after UL, it is generally accepted that the recovery of neuronal activity within the ipsi-VN plays a major role in behavioral recovery [2]. Moreover, other areas of CNS might also participate in VC. It has been reported that the cerebellum is involved in the neuronal recovery during VC [10–14]. The cerebellum is responsible for motor learning, and cerebellum cortex, especially the flocculus, contributes to vestibular adaptation [15]. The floccular target neuron (FTN) is localized at the rostral MVN and is involved in vestibular adaptation [16]. Fifteen to twenty percent of the neurons in the MVN are FTNs and receive monosynaptic inhabitation from the ipsilateral flocculus [11]. Moreover, the flocculus receives vestibular and visual afferents and the two inputs from the vestibular and visual afferents induce the changes in synaptic efficacy of Purkinje cells in flocculus [17]. The Purkinje cells in the flocculus regulate the function of the inhibitory vestibular-ocular neurons. Several studies indicate that the cerebellar flocculus-paraflocculus complex plays an important role in vestibular compensation [11,14]. Courjon *et al*. [13] reported that the ablation of the vestibulocerebellum, or the disruption of the climbing fibers input to flocculus, can severely delay the compensation. Flocculectomy after compensation following UL induces the reappearance of UL-induced acute labyrinthine dysfunction [12]. Therefore, it is suggested that both the cerebellum and MVN may play roles in the vestibular compensation process. On the other hand, during the precise time-course of “rebalancing” of neuronal activity in the bilateral vestibular nuclei, cellular, and molecular mechanisms involved this form of plasticity are yet to be elucidated.

The metabotropic glutamate receptors (mGluRs) have been shown to play a role with VC [18–20]. Electrophysiological studies have confirmed that MVN neurons in rats are endowed with mGluRs by the method of brainstem slices [20–22]. Compensatory increase of intrinsic excitability in ipsilesional MVN neurons is abolished by intra-floccular microinjections of the mGluR antagonist AIDA [11]. The mGluRs (mGluR1-8) have been further divided into three subgroups (groups I, II, and III) based on sequence similarities, ligand selectivity, and intracellular second messengers. Group-I consists of mGluR1 and mGluR5; Group-II of mGluR2 and mGluR3, and Group-III of mGluR4, mGluR6, mGluR7, and mGluR8 [23]. Biochemical studies indicated that activation of group II and III mGluRs suppresses glutamate release, whereas group I activation enhances glutamate release [23]. As a nonselective agonist of the glutamatergic metabotropic receptors, trans-1-amino-cyclopentane-1-3-dicarboxylic acid (*trans*-1-ACPD) can depolarize all the MVNs [19]. In contrast, monosynaptic excitatory post-synaptic potentials (EPSP) evoked in slices, by stimulating electrodes placed in the vestibular nerve, were inhibited by 1s-3R-ACPD [24]. This might reflect the ability of ACPD to activate certain group I, II, III receptor subtypes coupled to the predominant mGluR subtype expressing itself in that preparation or condition [23]. Among the mGluRs, mGluR1 is essential in synaptic plasticity such as long-term depression (LTD) in the Purkinje cells of the cerebellum [20,25]. At the presynaptic level, group II and III receptors are involved in negatively modulating of excitatory glutamate and/or inhibitory GABA neuronal transmission. Moreover, mGluR2 appear to be essential for inducing LTD at the mossy fiber CA3 synapse in the hippocampus [23,26]. The group II and III mGluRs in the hippocampus were shown to be localized exclusively at asymmetrical synapses (*i.e*., glutamatergic terminals) [26]. Of the Group-II and Group-III metabotropic receptor subtypes, mGluR2 receptors are localized to preterminal axons of neurons where they function as a negative feedback mechanism; mGluR7 receptors are widespread in distribution throughout the neuro-axis; mGluR3 receptors expressed in glia where their functional role is less clear; mGluR4 and mGluR8 receptors are restricted in distribution in the central nervous system; mGluR6 receptors have been shown in the retina [26]. Recently, it was reported that activation of postsynaptic mGluR7 significantly reduced NMDAR-mediated currents, causing the internalization of NMDARs [27]. Therefore, the mGluR2 and mGluR7 were chosen because they are important subunits for Group-II and III of mGluRs, respectively [26,27]. The expression of mGluR2 and mGluR7 mRNA, or protein in the VNC and cerebellum, has been confirmed by *in situ* hybridization and immunohistochemical methods [28,29]. In addition, mGluR7 mRNA expression was downregulated in the ipsilateral VNC at 6 h following UL in rats using real-time quantitative PCR, and it was found that no changes of mGluR7 mRNA in either the flocculus or inferior olive occurred [20]. However, the current molecular evidence relating to mGluRs expression has been restricted to studies of mRNA expression, and it is possible that the levels of protein expression different from the corresponding mRNA expression. Until now, no studies have examined the protein expression of mGluRs in the MVN and flocculus after UL. The aim of this study was to investigate the potential role of mGluRs in the MVN and flocculus in different stages following UL in rats.

## 2. Results and Discussion

### 2.1. Behavior Observation after UL

**Figure 1:**
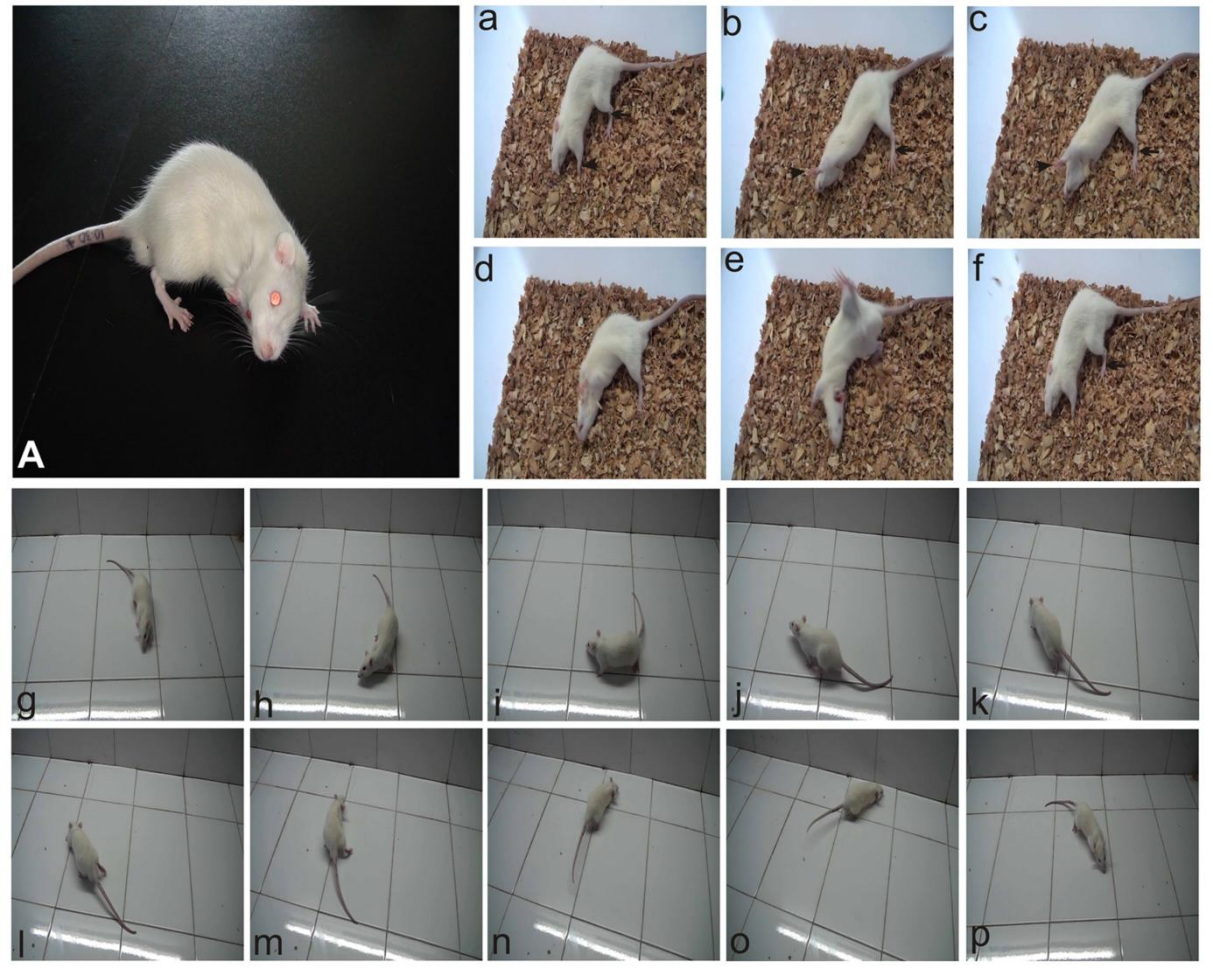
Behavioral aymmetries after unilateral labyrinthectomy on the right side. (**A**) Demonstrated the head tilting towards the operated side; (**a**–**f**) and (**g**–**p**) showed the rolling and circling movements, respectively. Note head rotation and the displacement of forelimb (arrowheads) and hind limb (arrows) on the intact side (left side in this case).

The manifestation due to acute labyrinthine dysfunction such as head tilting toward the operated side, limb extending on the intact side (Figure 1A), body spinning (Figure 1a–f), and circling walk (Figure 1g–p) were observed in all rats at the 1st day after UL. The static symptoms, such as spontaneous nystagmus (SN) and postural asymmetry were severe within the 1st day post surgery and relieved progressively over time until the 3rd day. As expected, the sham-operated rats did not show any ocular motor or postural deficits.

### 2.2. UL Induces the Changes of mRNA Levels of mGluR2 and mGluR7 in the MVN and Flocculus

**Figure 2:**
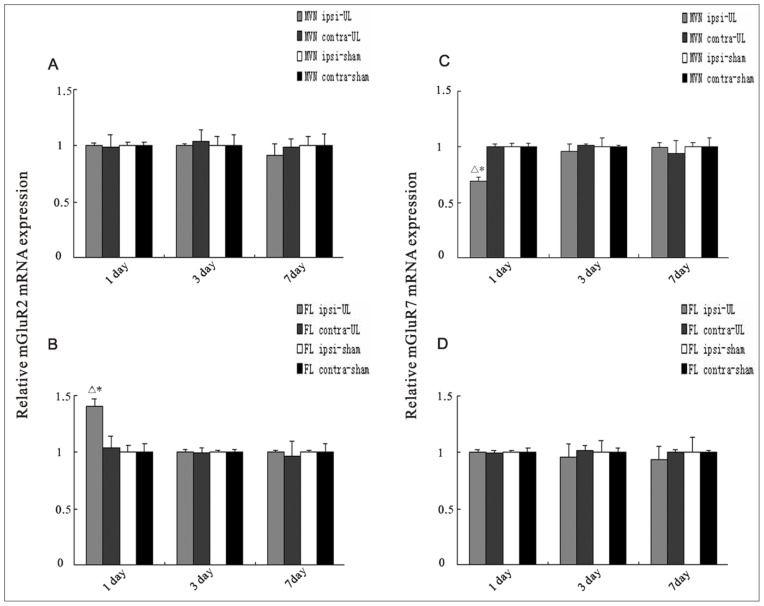
Quantitative analysis of the mRNA expression of mGluR2 (**A**,**B**) and mGluR7 (**C**,**D**) in the MVN and flocculus at the 1st, 3rd, and 7th day following UL or sham operation. The mRNA level of mGluR2 was significantly increased in the ipsilateral (ipsi) flocculus at the 1st day after UL compared to sham controls and contralateral (contra) side. The columns represent means ± SEM of six rats per group. * *p* < 0.01 *vs*. 1 day post sham; ^Δ^*p* < 0.05 *vs*. contra. The mRNA level of mGluR7 was significantly decreased in the ipsilateral (ipsi) MVN at the 1st day after UL compared to sham controls and contralateral (contra) side. Columns represent means ± SEM of six rats per group. * *p* < 0.05 *vs*. 1 day post sham; ^Δ^*p* < 0.01 *vs*. contra.

There were no changes in the expression of mGluR2 mRNA in the MVN cross time after UL (see Figure 2A). However, mGluR2 mRNA expression was increased in the ipsilateral flocculus at the 1st day after UL, compared to the sham controls (*p* < 0.01) and the contralateral side (*p* < 0.05) (see Figure 2B). This difference was not observed by either the 3rd or 7th day (see Figure 2B). By contrast, the contralateral flocculus and the sham controls have no related changes of the mRNA expression (see Figure 2B).

There were no changes in the mRNA expression of mGluR7 in the flocculus cross time after UL (see Figure 2D). However, the mRNA expression of mGluR7 in the ipsilateral MVN (see Figure 2C) was significantly reduced compared to the contralateral MVN (*p* < 0.01) and sham controls (*p* < 0.05) at the 1st day after UL. This difference had also disappeared by either the 3rd or 7th day (see Figure 2C). By contrast, the contralateral MVN and the sham controls have no related changes of the mRNA expression (see Figure 2C).

### 2.3. UL Induces the Changes of Protein Levels of mGluR2 and mGluR7 in the MVN and Flocculus

**Figure 3:**
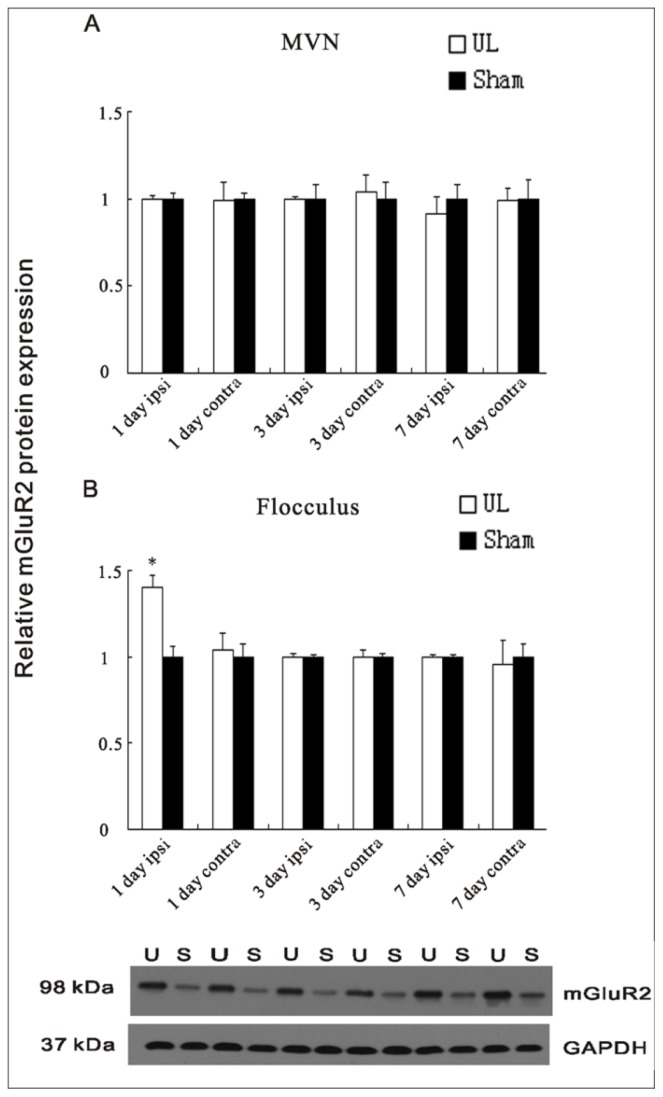
Quantification of the mGluR2 protein expression in the ipsilateral (ipsi) and contralateral (contra) MVNs (**A**) and flocculus (**B**) at the 1st, 3rd, and 7th day following UL compared to sham operation at the same time; (**B**) The protein level of mGluR2 was increased in the ipsilateral (ipsi) flocculus at the 1st day after UL compared to the ipsilateral (ipsi) sham controls. The columns represent means ± SEM of six rats per group. Inset shows a Western blot for mGluR2 in the ipsilateral flocculus at the 1st day following UL compared to sham controls at the 1st day postoperative. Ten micrograms of protein were applied in each lane (*n* = 6 for both UL and sham groups). U = UL, S = sham. * indicates a significant difference from ipsilateral sham controls (*p* < 0.01).

The mGluR2 protein expression did not change over time after UL in the MVN (see Figure 3A). However, the expression of mGluR2 protein in the flocculus significantly increased on the ipsilateral side at the 1st day after UL (*p* < 0.01; see Figure 3B) compared to the sham controls, while at the 3rd day and 7th day after UL, there was no difference in protein expression (see Figure 3B). By contrast, the contralateral flocculus and the sham controls have no related changes (see Figure 3B).

**Figure 4:**
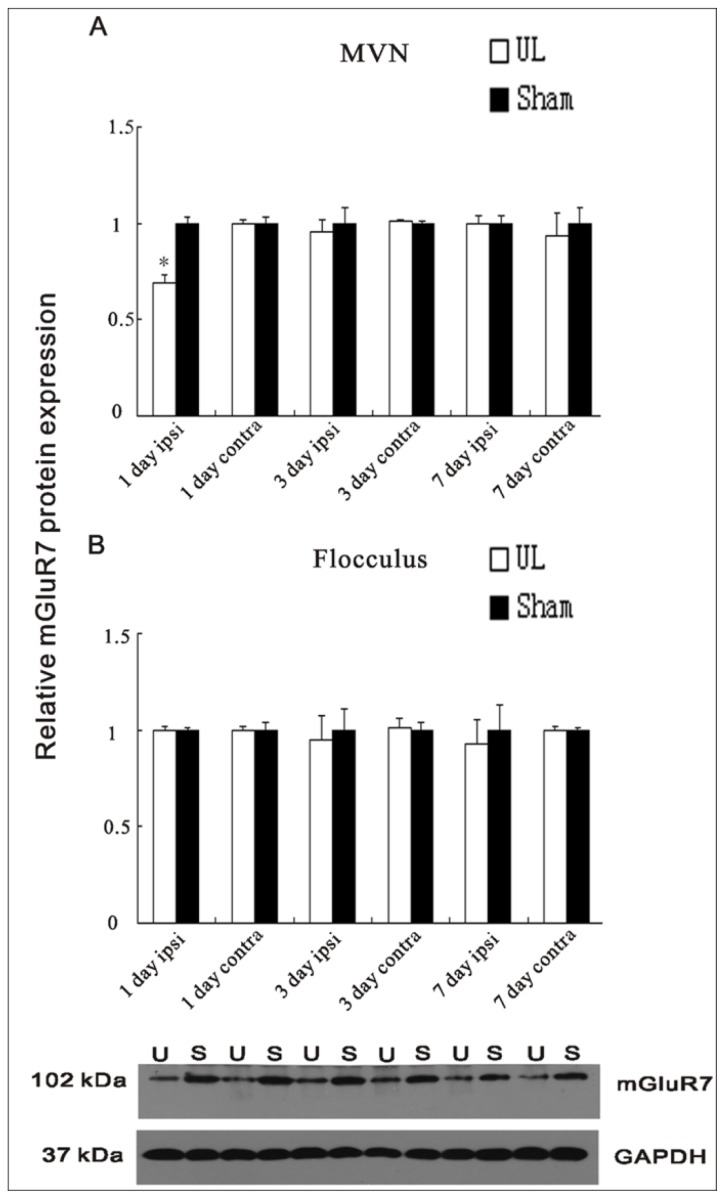
Quantification of the mGluR7 protein expression in the ipsilateral (ipsi) and contralateral (contra) MVNs (**A**) and flocculus (**B**) at the 1st, 3rd, and 7th day following UL compared to sham operation at the same time; (**A**) The protein level of mGluR7 was decreased in the ipsilateral MVN at the 1st day after UL compared to the ipsilateral sham controls. The columns represent means ± SEM of six rats per group. Inset shows a Western blot for mGluR7 in the ipsilateral MVN at the 1st day following UL compared to sham controls at the 1st day postoperative. Ten micrograms of protein were applied in each lane (*n* = 6 for both UL and sham groups). U = UL, S = sham. * indicates a significant difference from ipsilateral sham controls (*p* < 0.01).

There were no changes in protein expression of mGluR7 in the flocculus over time after UL (see Figure 4B). However, the protein level of the mGluR7, in the ipsilateral MVN, decreased at the 1st day after UL (*p* < 0.01; see Figure 4A), compared to the sham controls, while at the 3rd day and 7th day after UL, there was no difference in protein expression (see Figure 4A). By contrast, the contralateral MVN and the sham controls have no related changes (see Figure 4A).

### 2.4. UL Induces the Asymmetrical Distribution of mGluR2 and mGluR7 in Bilateral MVN and Flocculus

**Figure 5:**
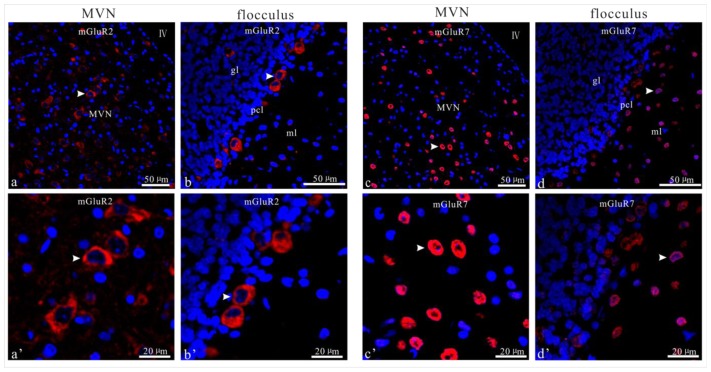
Illustration of mGluR2 and mGluR7 immunoreactive neurons in the MVN and flocculus of sham control rats. (**a**,**b**,**a**′,**b**′) showed the mGluR2 in the MVN and flocculus; (**c**,**d**,**c**′,**d**′) showed the mGluR7 immunolabled neurons in the MVN and flocculus; (**a**′–**d**′) are the enlarged cell images in (**a**–**d**). The same cells can be identified by the arrowheads.

In the control rats, the presence of mGluR2 and mGluR7 in the MVN and flocculus were shown by immunostaining (see Figure 5). The expression of mGluR2 and mGluR7 were distributed in the neurons throughout the MVN. In the flocculus, the mGluR2 positive neurons were concentrated in the granule cell and Purkinje cell layer; the mGluR7 positive neurons were concentrated in the molecular and Purkinje cell layers (Figure 5).

**Figure 6:**
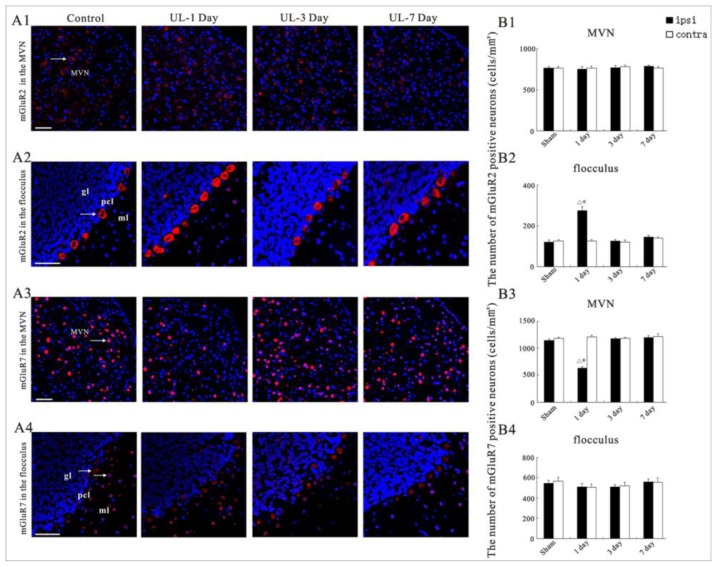
(**A1**–**A4**) Confocal images showing the expression of the mGluR2 and mGluR7 in the same area of the ipsi-lesional MVN and in the same area of the ipsi-lesional flocculus following UL or sham controls at different postoperative days. Arrows show the mGluR2- and mGluR7-positive neurons. UL-1 Day: the 1st after UL, UL-3 Day: the 3rd after UL, UL-7 Day: the 7th day after UL, Control: with intact labyrinths; MVN: medial vestibular nucleus; gl: granule layer; pcl: Purkinje cell layer; ml: molecular layer. Calibration bar: 50 μm; (**B1**–**B4**) Quantitative evaluation of the effects of a UL on mGluR2 and mGluR7 immunoreactive neurons in the MVN and flocculus. The number of mGluR2 and mGluR7-positive neurons in each corresponding region of the visual system was expressed per unit area (mm^2^). Columns represent means ± SEM. The values recorded on the ipsilateral (black columns) and contralateral (white columns) sides are given separately for all rats. The data from the subgroups of sham rats (the 1st, 3rd, and 7th day after sham operation) were pooled to provide a direct comparison with the subgroups of rats sacrificed at the 1st, 3rd, and 7th day after UL. * *p* < 0.01 *vs*. 1 day post sham; ^Δ^*p* < 0.01 *vs*. contra.

In the UL rats, at the 1st day after UL, the mGluR7 staining in the MVN decreased on the ipsilateral side compared to sham controls, but the mGluR7 staining on the contralateral side did not change compared to sham controls. At the 3rd day after UL, the mGluR7 staining on both sides of the MVN was similar to that of the sham controls. At the 7th day after UL, the mGluR7 staining on both sides of the MVN was similar to the postoperative day three (Figure 6A3,B3). However, in the MVN, there were no changes in the number of neurons expressing mGluR2 over time after UL (Figure 6A1,B1). At the 1st day after UL, in the flocculus the mGluR2 staining increased on the ipsilateral side compared to sham controls, whereas mGluR2 staining on the contralateral side did not change compared to sham controls. At the 3rd day after UL, the mGluR2 staining in both sides of the flocculus was similar to that of the sham controls. At the 7th day after UL, on both sides of the flocculus, the mGluR2 staining was similar to the postoperative day three (Figure 6A2,B2). However, in the flocculus, there were no changes in the number of neurons expressing mGluR7 over time after UL (Figure 6A4,B4).

### 2.5. Discussion

#### 2.5.1. Medial Vestibular Nucleus

The MVN is an important area of brain involved in vestibular compensation. Electrophysiological evidences have shown that the recovery of neuronal activity in the ipsilateral MVN after UL might play a key role in the compensation process [2,30]. Therefore, to understand the mechanisms of lesion-induced plasticity in the CNS, it is essential to examine the molecular and biochemical changes during recovery of neuronal activity.

Our study evaluated change pattern in mGluR2 and mGluR7 protein in the MVN after UL. The present study demonstrated mGluR2 expression in the MVN after UL did not change at either the mRNA or protein levels. This was true for both the ipsilateral and contralateral MVN at each post-operative time, when compared with sham controls. In each case, mRNA and protein from the UL and sham control groups were analyzed within the same plates and same gels, thereby eliminating potential errors that could be introduced by comparing between different plates and gels. By contrast, the mRNA and protein expression of mGluR7 decreased in the ipsilateral MVN at the 1st day after UL compared to both sham controls and the contralateral MVN. These differences had disappeared by three and seven days after UL. Horri *et al*. [20], using real time PCR in rats, confirmed that mGluR7 mRNA expression decreased in the ipsilateral VNC at 6 h, which recovered to control levels by 50 h after UL. Their results support the conclusion that mGluR7 is down-regulated in the ipsilateral VNC following UL. The present results agree with this view, and indeed serve to emphasize the key role that both the mRNA and protein levels had the same change pattern. Ris *et al*. [31,32] reported that, during seven days after UL, the normal resting discharge recovery of the ipsilateral MVN neurons plays a crucial role in the disappearance of the static syndrome through rebalancing activity of bilateral vestibular nuclei neurons. In this study, the acute labyrinthine dysfunction was occurred in all rats at the 1st day after UL and static symptoms partially disappeared at the 3rd day after UL. The observations of animals’ behaviors, after UL, were in agreement with the previous report [33]. The mGluRs have been reported to be involved in long term potential and LTD. These receptors have been demonstrated to affect many MVN neurons, either by increasing or decreasing their firing rate [5,21]. These findings suggested that decrease of mGluR7 after UL in the ipsilateral, but not in the contralateral, MVN is correlated with the hypoactivity of ipsilateral afferent nerves after UL. It is possible that the recovery of mGluR7 expression at the 3rd day post-UL in the ipsilateral MVN may be related to the recovery of the behavioral symptoms and neuronal activities in the MVN.

Several studies have demonstrated that mGluR7 is predominantly localized at presynaptic elements in the dorsal horn and dorsal root ganglion of the hippocampus and basal ganglia [29,34,35]. Among the mGluRs, only mGluR7 is located at the active zone of the presynaptic terminal [23]. Perhaps the most well characterized effect of mGluR7 is the inhibition of glutamate release as autoreceptor. However, Gu *et al*. [27] reported that activation of postsynaptic mGluR7 significantly reduced NMDAR-mediated currents. The mGluR7 distributes not only in the glutamatergic synaptic terminals, but also in non-glutamatergic neurons, such as the GABAergic terminals [23,36]. Previous data confirmed that L-AP4, a group III mGluR agonist, can inhibit GABA releasing by activating mGluR7 in cultured mouse striatal GABAergic neurons [26]. Therefore, presynaptic and/or postsynaptic mGluR7 may regulate the recovery of behavioral symptoms and resting activity in the ipsilateral MVN during VC.

#### 2.5.2. Flocculus

The cerebellum appears to be important in the initial recovery from static symptoms after UL. The ipsilateral flocculus is necessary to increase the intrinsic excitability of MVN neurons after UL [11–13,37–40].

The present study demonstrated that mGluR7 expression in the flocculus after UL did not change at either the mRNA or protein levels. Using RT-PCR, Horii *et al*. [20] also found that no changes in mGluR7 mRNA expression were observed in the flocculus at either 6 h or 50 h after UL. It is possible that the VC process is not associated with a regulation of mGluR7 in the flocculus or the mGluR7 function does change in the flocculus during VC as a result of modifications in affinity or efficacy rather than changes in the levels of mGluR7 mRNA or protein. However, there have been no reports available describing changes in mGluR2 in the flocculus after UL at either the mRNA or protein levels. The present results demonstrated that mGluR2 expression at both the mRNA and protein levels increased in the ipsilateral flocculus at the 1st day after UL compared to both sham controls and the contralateral flocculus. Our immunohistochemical results agree with other studies [41,42] that described in the flocculus, mGluR2 present in the granule cell layer and Purkinje layer. They demonstrated that mGluR2 present in the cell body of the granular layer (dendrites and axon terminals of Golgi cells). It is probable that in Golgi cell the presynaptic mGluR is mGluR2. Golgi cell are GABAergic neurons. Spillover of glutamate from mossy fiber terminals can activate presynaptic mGluR2 in Golgi cell-granule cell synapses and this leads to a suppression of Golgi cell inhibition. Consequently, facilitates transmission of the mossy fiber input to the parallel fiber system. And then spillover of glutamate from the facilitated parallel fiber terminals can activate mGluR1 in Purkinje cells [26,43,44]. Hartell [45] demonstrated in cerebellar slices that the mGluRs (especially the mGluR1) were necessary for LTD at synapses between parallel fibers and Purkinje cells. Therefore, mGluR2 can play a role in LTD of parallel fibers and Purkinje cells synapses. The Purkinje cell is a major type of inhibitory neuron that releases GABA, and GABA could be the neurotransmitter involved in the direct cerebello-vestibular pathways [46]. In addition, Johnston *et al*. [11] reported that cerebellar cortical plasticity after UL, involving the expression of LTD in the ipsi-lesional flocculus, leads to the induction of compensatory increase in intrinsic excitability in MVN neurons. Taken together, these findings and our data demonstrate that an increase in mGluR2 expression after UL in the ipsilateral flocculus, might enhance LTD in the ipsilateral flocculus. This might result in a decrease in floccular inhibition to the ipsilateral MVN neurons and help to rebalance the resting activity between the ipsilateral and contralateral MVNs.

## 3. Experimental Section

### 3.1. Animal Procedures

Data were obtained from a total of 90 male Sprague-Dawley rats (200–250 g). Thirty-six animals were used for the Western blotting study, 36 were used for quantitiative real-time PCR study, and 18 were used for the immunohistochemical study. The animals were obtained from the experimental animal center of Tongji Medical College, Huazhong University of Science and Technology. The animals had free access to food and water. All experimental procedures were conducted following the National Institutes of Health “Guide for the Care and Use of Laboratory Animals” (NIH Publications No. 80-23, revised 1996) and were approved by the College’s Committee on Animal Research. Experimental animals for Western blotting or quantitiative real-time PCR were randomly divided into sham controls (*n* = 18) and experimental (*n* = 18) groups. Animals were sacrificed at the 1st, 3rd, or 7th day after a right UL or right sham operation. We examined changes in mRNA and protein expressions at the 1st day after UL as it represents the uncompensated stage of vestibular compensation in rats, when SN is vigorous and postural asymmetry is severe. These symptoms partially disappear by the 3rd day after UL and by the 7th day after UL, these symptoms have substantially compensated but the dynamic reflex deficits remain. Therefore, these times were chosen as representative time points for the compensated stage [14,47,48].

### 3.2. Unilateral Labyrinthectomy

For the UL group, the labyrinthectomy was performed on the right side under an operating microscope after the rats were anesthetized with ketamine (2 mL/kg) and chlorpromazine (0.2 mL/kg); 1 mL of xylocaine (with 1:10,000 adrenaline) was used in the wound margin. Firstly, the right tympanic bulla was opened by a retroauricular surgical approach. After removing the malleus and incus, the vestibule was visualized. The stapedial artery was cauterized at two points. Secondly, a small hole was made around the oval window using the surgical electrodrill and the membranous labyrinth was removed by aspirating with a suction pump. Finally, absolute alcohol was injected into the small opening to ensure the vestibule was completely destroyed. At the end of the surgery, the wound was sutured. Neither the pterygopalatine artery nor the facial nerve was damaged by these procedures. This method for the labyrinthectomy has been established by Dr T. Kitahara [49]. Histological studies confirmed that the labyrinth was completely damaged and the lesion did not extend to the adjacent brain [49]. The animals recovered from the anesthesia before 4 h post-UL. The animals were housed in individual cages after UL. For the sham operation group, animals had their right the retroauricular skin incised in the same way as for the UL. The tympanic bulla was only opened without the destruction of tympanic membranous and ossicles after exposed. Other procedures were the same as for the UL group.

### 3.3. Quantitative RT-PCR

After decapitation, the bilateral flocculus and paraflocculus were carefully removed from the skull. The brains were immediately placed into 0.9% ice-cold saline for 1 min. Following the rat brain atlas of Paxinos and Watson [50], horizontal brainstem slices, including the MVN, were taken in order to dissect each nucleus. To take a slice including the MVN, a brainstem slice (1.5 mm thickness) just caudal from the abducens nucleus was dissected, and the bilateral MVN were dissected separately under microscopic guidance using a sharp blade [20]. All these procedures were performed on a chilled plate in order to prevent possible RNA degradation.

Total RNA was extracted using an RNeasy mini kit (Axygen, Hangzhou, China) according to the manufacturer’s instructions. cDNA was reverse transcribed using a PrimeScript RT reagent Kit with gDNA Eraser (TaKaRa, Dalian, China). The RNA and cDNA of each sample were analyzed using a GeneQuant pro DNA/RNA calculator to assess the concentrations and purity. The cDNA samples were stored at −20 °C until use. Quantitative real-time PCR was performed by applying the real-time SYBR Green PCR technology with the use of a 7300 Real-time PCR System (Applied Biosystems, Foster City, CA, USA). Validated primers were designed for each target mRNA. The primer pairs for mGluR2, mGluR7, and an internal standard (β-actin) were as follows: mGluR2 forward, 5′-TGGTGGCTCCTACAGTGTCTC-3′; mGluR2 reverse, 5′-TAACGGGACTTGTCACTCAGCTTG-3′; mGluR7 forward, 5′-CCTTGCTGCTGGACCTGTGA-3′; mGluR7 reverse, 5′-CTGGTCGTAGGGACAATGCTGA-3′; β-actin forward, 5′-CCTGGAGAAGAGCTATGAGC-3′; β-actin reverse, 5′-ACAGGATTCCATACCCAGG-3′. The amplification conditions were as follows: 1 min at 95 °C, and then 40 cycles of 15 s at 95 °C, 20 s at 60 °C, and 35 s at 72 °C. An internal standard was used to normalize the relative gene expression levels. A melting curve analysis was performed for each gene, and the specificity and integrity of the PCR products were confirmed by the presence of a single peak. The relative expression was calculated from the differences in the Ct values between the target mRNA and an internal standard (β-actin). The change in the relative mRNA levels between the ipsilateral and contralateral UL group or UL group and the sham group were analyzed by using the 2^−ΔΔ^*^Ct^* method, as previously reported [51].

### 3.4. Western Blotting

After decapitation, the bilateral flocculus and paraflocculus were carefully removed from the skull. The brains were immediately placed into 0.9% ice-cold saline for 1 min. Following the rat brain atlas of Paxinos and Watson [50], the left and right MVNs were dissected under a microscope using the detailed procedures described previously by Horri *et al*. [20]. Protein of the MVN and flocculus was extracted respectively. Protein concentrations were determined using the BCA Protein Assay Kit (Pierce Biotech Inc., Rockford, IL, USA). The sample from one animal was loaded to one lane without mixture of tissues from other animals. Ten micrograms of each sample from the MVN and flocculus of one animal were loaded in each lane in sample buffer, separated on 8% SDS-PAGE gel. After electrophoresis, the SDS gel was trimmed into slim bands containing target proteins according to the Prestained protein markers (10–170 kDa; Bio-Rad, Hercules, CA, USA) running in both sides of the gel as marginal lanes with enough space on both trimmed edges of target proteins bands. The rest gel out of these trimmed target bands was abandoned. Comparisons between UL and sham groups were always made on the same gels and all of the assays were repeated to confirm their accuracy [33,52,53]. Then, the target proteins in the trimmed SDS gel were transferred onto PVDF membranes using a transblotting apparatus (Bio-Rad, Hercules, CA, USA) for 1.5 h (mGluR2, 98 kDa; mGluR7, 102 kDa) in transfer buffer containing 20% methanol, 1.5% glycine, and 0.3% Tris-base. The blots were blocked with 5% non-fat milk in TBS for 1 h. The immune-detection on the Western blot was carried out with affinity-purified polyclonal antibodies raised against the mGluR2 and mGluR7 (abcam, Cambridge, UK) diluted at a dilution of 1:1000. The antibody of mGluR2 reacted with mGluR2 protein at 98 kDa and the antibody of mGluR7 reacted with mGluR7 protein at 102 kDa, respectively. Secondary anti-rabbit antibodies were applied at a dilution of 1:3000. Membranes were visualized using ECL plus (Pierce Biotech Inc., Rockford, IL, USA). Films were analyzed by densitometry to determine the quantity of protein expressed in each group using the Bio-Rad Quantity One software (Bio-Rad, Hercules, CA, USA). GAPDH was used as an internal control. Results were expressed as optical density [52,54].

### 3.5. Immunohistochemistry

Following the completion of the quantitative real-time PCR and Western blotting study, the aim of this experiment was to use immunohistochemistry to label neurons with mGluR2 and mGluR7 in the MVN and in the flocculus, and then determine whether the number of neurons expressing these subunits changed after UL. Animals were randomly divided into six groups of three animals: sham surgery controls and the UL groups at the 1st, 3rd, or 7th day after UL. The animals were anesthetized with ketamine (2 mL/kg) and chlorpromazine (0.2 mL/kg), and subsequently perfused transcardially with fixative (4% paraformaldehyde in 0.1 M phosphate buffer) following a wash through with 0.9% normal saline. After decapitating the bodies, the temporal bones were removed and brains were dissected out. The cerebellum and brain stem portion were fixed in the same fixative for at 12 h at 4 °C and then rinsed with distilled water for half an hour, dehydrated with a graded alcohol series, cleared in xylene, immersed in paraffin, and then embedded in paraffin. Finally, the brainstems with the flocculus were sectioned at 5 μm, and collected on poly-l-lysine-coated glass slides glass slide. The boundaries of the MVN were identified based on the brain atlas by Paxinos and Watson [50]. Four sections from the rostral to the caudal level ((Bregma −9.96 mm)–(Bregma −12.00 mm)) from each rat in the MVN or flocculus were collected throughout the MVNs and flocculus. Two sections were selected from the rostral and the caudal part of the brain which including the MVN or fland the ((Bregma −9.96 mm)–(Bregma −12.00 mm)) using the guidance of the atlas represented the rostral and caudal zones of Purkinje cell/climbing fiber zones, which control the eye movement in all vertical planes from sagittal to transverse planes [55]. Two sections at 50 μm intervals were selected from the middle part of the brain which including the MVN or flocculus ((Bregma −9.96 mm)–(Bregma −12.00 mm)) using the guidance of the atlas represented the middle zone of Purkinje cell/climbing fiber zones, which controls the eye movement in the horizontal plane [56–58].

After deparaffinized in xylene and rehydrated through graded concentrations of ethanol, two series of the brainstem sections with flocculus were incubated in primary antibody raised against the mGluR2 and mGluR7 (abcam, Cambridge, UK) in blocking solution overnight at 4 °C, respectively. The tissue was washed and incubated in fluorescence-labeled secondary antibody (Alexa Fluor 555, Invitrogen/Molecular Probes, Carlsbad, CA, USA) for 1 h at room temperature. After being washed, the nuclei were counterstained with a DAPI staining solution (Beyotime, Haimen, Jiangsu, China) for 5 min at room temperature. After being washed, the sections were examined under a laser scanning confocal microscope (Nikon, Tokyo, Japan). Control tests were done by incubating the specimen with PBS instead of the primary antibody and no immunostaining was observed. Adobe Photoshop 5.0 (Adobe Software, San Jose, CA, USA) was used to adjust the size and brightness of the images during the preparation of the figures [59]. The number of mGluR2 and mGluR7 positive neurons in each corresponding region of the visual system in the same area of the bilateral MVN and the bilateral flocculus were counted, with the number of labeled neurons expressed per unit area (mm^2^) [60–62]. Neurons were counted only if their nuclei were completely within the margins of the visual system by using Image-Pro-Plus (6.0) software (Media Cybernetics, Bethesda, MD, USA). All images were acquired using the same exposure time and illumination conditions. Histological quantification was conducted by an investigator blinded to the experimental status of the animals.

### 3.6. Statistical Analysis

Data are presented as mean ± SEM. The analysis was performed with SPSS 13.0 software (SPSS Inc., Chicago, IL, USA). Statistical significance was tested with a one-way analysis of variance followed by Student Newman–Keuls multiple comparison tests or non-paired Student’s *t*-test. *p* values under 0.05 were considered significant.

## 4. Conclusions

**Figure 7:**
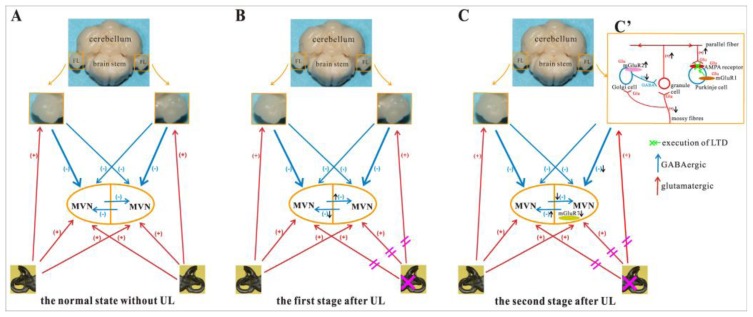
Schematic illustration for mGluR2 and mGluR7 mediated regulation in the MVN and flocculus during vestibular compensation. (**A**) the normal state without UL: The resting activities of bilateral MVN neurons are normal and balanced; (**B**) the first stage after UL: Immediately after UL, the deafferented ipsilateral MVN neurons lose their normally high resting activity, while the contralateral neurons become hyperactive; (**C**) the second stage after UL: The downregulation of mGluR7 in ipsi-lesional MVN and upregulation of mGluR2 in ipsi-lesional flocculus may play a role in rebalancing of resting activity between the ipsilateral and contralateral MVNs; (**C**′) the role of mGluR2 in flocculus after UL: upregulation of mGluR2 can suppress Golgi cell inhibition. This facilitates transmission of the mossy fiber input to the parallel fiber system. The facilitated parallel fiber stimulation causes the release of a sufficient amount of glutamate to activate mGluR1. Then, the mGluR1 induces parallel fiber-Purkinje cell LTD. FL: flocculus; Glu: Glutamate; LTD: long-term depression; MVN: medial vestibular nucleus.

In conclusion, we investigated the changes of both the mRNA and the protein expression of mGluR2 and mGluR7 in the MVN and the flocculus at the 1st, the 3rd, and the 7th day following UL. Our results have demonstrated that mGluR7 was down-regulated in the ipsilateral MVN at the 1st day after UL; the mGluR2 was up-regulated in the ipsilateral flocculus at the 1st day after UL. Our results suggest that the changes of mGluR2 and mGluR7 expression might play a role in rebalancing of resting activity between the ipsilateral and contralateral MVNs (Figure 7). Furthermore, we would have to determine how to change for the mGluR1 in the flocculus during the process of vestibular compensation; which type of specific neurons with mGluR2 or mGluR7 expression contributes to the vestibular compensation and how do these neurons work in the vestibulocerebellar system.
